# Physical Health and Socioeconomic Status in Ambulatory Adults With Bilateral Spastic Cerebral Palsy

**DOI:** 10.1155/2024/8368191

**Published:** 2024-10-29

**Authors:** Maaike M. Eken, Nelleke G. Langerak, Jacques du Toit, Melanie Saywood, Robert P. Lamberts

**Affiliations:** ^1^Division of Orthopaedic Surgery, Department of Surgical Sciences, Faculty of Medicine and Health Sciences, Stellenbosch University, Tygerberg, South Africa; ^2^Division of Sport and Exercise Medicine, Department of Exercise, Sport and Lifestyle Medicine, Faculty of Medicine and Health Sciences, Stellenbosch University, Tygerberg, South Africa; ^3^Neuroscience Institute and Division of Neurosurgery, Department of Surgery, Faculty of Health Sciences, University of Cape Town, Cape Town, South Africa; ^4^Department of Research, Sint Maartenskliniek, Nijmegen, The Netherlands; ^5^Orthopaedic, Sports and Rehabilitation Center, Linksfield Hospital, Johannesburg, South Africa; ^6^Division of Movement Science and Exercise Therapy (MSET), Department of Exercise, Sport and Lifestyle Medicine, Faculty of Medicine and Health Sciences, Stellenbosch University, Stellenbosch, South Africa

**Keywords:** balance, cerebral palsy, spasticity

## Abstract

Socioeconomic status (SES) tends to influence an individual's access to health care. It is commonly assumed that a poorer SES is associated with a weaker physical health status, especially in disadvantaged populations such as people with cerebral palsy (CP). However, to our knowledge, no study has looked at this assumption. Therefore, the aim of this study was to describe and compare the physical health status of ambulant adults with bilateral CP with different SES backgrounds. In addition, the physical health status of the ambulatory adults with CP was compared to well-matched, typically developing adults. Twenty-eight ambulatory adults with CP (gross motor functional classification system Level I/II/III: *n* = 11/12/5; SES low/middle/high: *n* = 10/9/9), and 28 matched typically developing adults were recruited for this study. No differences were observed between adults with CP from different SES backgrounds. Differences in physical health status between typically developing adults and ambulatory adults with CP in all SES backgrounds were found in passive range of motion (*p* < 0.05), muscle strength (*p* < 0.001), selectivity (*p* < 0.001), and muscle tone (*p* < 0.001) and balance (*p* < 0.05). The main finding of this study is that physical health status did not differ between ambulatory adults with CP from different SES backgrounds. This finding shows that SES does not always directly impact physical health status in ambulatory adults with CP and highlights the importance of an individual approach. Future research should determine the impact of SES on nonambulatory adults with CP.

## 1. Introduction

Cerebral palsy (CP) is widely recognized as a common childhood-onset physical disability. Recent research shows that more than 80% of adults with CP nowadays have a life expectancy of more than 58 years [1]. Markedly improved survival rates among adults with CP have been reported, especially in those with minor disabilities classified in Gross Motor Function Classification System (GMFCS) Levels I, II, and III [2]. As a result of these positive developments, the adult population with CP is currently growing.

Over the past decades, the number of adults with CP attending rehabilitation practices has, thus, increased, along with the amount of research data being generated [3]. Although the lesion in the brain that causes CP is nonprogressive, secondary problems in physical status and balance control tend to develop during childhood and adolescence and could progress as individuals with CP grow into adulthood [4]. As a result, ageing with CP is associated with certain limitations pertaining to health and functioning.

Previous studies have shown that passive range of motion (PROM) [5], muscle strength [6], and balance [7] are often compromised in individuals with CP when compared to typically developing (TD) adults. A combination of the abovementioned factors can result in impairments such as muscle contractures and bony deformities, as well as secondary problems such as impaired balance control.

Previous research has shown a worldwide prevalence rate of 2–3 per 1000 live births [8], while in low-to-middle-income countries, such as South Africa, the prevalence of CP has been estimated to be as high as 2–10 per 1000 live births [9]. Nevertheless, the majority of research has been conducted in high-income countries, while limited studies focus on CP adults living in low-to-middle-income countries. Adults with CP living in low-to-middle-income countries, when compared to those residing in high-income countries, have to navigate many variables related to socioeconomic status (SES) (including living conditions, environmental factors as well as problematic access to medical care, physiotherapy, assistive devices, and transport), which often result in unique secondary sequela [10]. Previous research has emphasized that environmental factors can influence physical activity and participation in mobile and leisure activities [11]. Yet, the influence of SES on physical status among adults with CP living in low-to-middle-income countries remains unclear. Therefore, the main aim of this study was to investigate possible differences in the physical health status of ambulant adults with bilateral spastic CP (from low, middle, or high SES). The secondary aim of this study was to quantify the magnitude of physical health status differences between adults with bilateral spastic CP and matched TD adults from similar SES backgrounds.

## 2. Materials and Methods

### 2.1. Participants

Ambulant adults with bilateral spastic CP were recruited for the study. Inclusion criteria for the study were: a diagnosis of bilateral spastic CP (with or without mild upper extremity involvement) and being able to walk (with or without assistive devices, i.e., GMFCS Levels I, II, or III) [12]. All adults received an interval surgery approach (ISA) during childhood [10, 13], while individuals who underwent a neurosurgical intervention (such as selective dorsal rhizotomy (SDR)), as well as adults who were diagnosed with other types of CP (e.g., athetoid, ataxic or mixed), were excluded from this study. TD adults were recruited via friends and family from adults with CP who were living in similar conditions and included when matched for age, sex, and body mass index (BMI). The study was approved by local institutions and conducted according to principles set out by the Declaration of Helsinki [14]. All participants provided written informed consent before enrolling in the study. The following outcomes were assessed to describe physical health status: PROM, lower limb strength, selectivity, muscle tone, and balance.

### 2.2. Assessment Procedures

Participants' age, sex, current health status, and SES were recorded during a structured interview. As the probing of income levels was deemed unethical by the local ethics committee, *housing density* was used as a predictor of SES [15]. This method, which has been shown to be valid and reliable in a South African setting, predicts SES based on the number of people living in the house divided by the number of rooms within the house (excluding the kitchen and bathroom) [15]. Consequently and based on the score, participants were either as having a low SES, > 1.5; middle SES, 1.0–1.5; or high SES, < 1.0. In addition to SES, the adults with CP were also classified based on the GMFCS level (either as I, II, or III) [12]. As part of the physical health screening, height and weight were measured, while BMI was calculated, and the total number and type of orthopaedic interventions received during childhood were obtained from detailed medical records.

#### 2.2.1. Range of Motion

The physical assessments of all participants were performed by the same paediatric orthopaedic surgeon (specialized in CP), and the physical assessment guidelines of Novacheck, Trost, and Sohrweide were followed [16]. PROM was measured using a goniometer. These measurements included the following joint angles: hip flexion, hip extension, hip abduction, hip adduction, hip external rotation, hip internal rotation, femoral anteversion, knee flexion, knee extension, unilateral popliteal angle, bilateral popliteal angle, hamstring shift, thigh foot angle, bimalleolar axis, ankle dorsiflexion with knee flexed, ankle dorsiflexion with extended knee, and ankle plantar flexion. Good intra and intersession test and retest reliability were observed in individuals with CP [17].

#### 2.2.2. Strength

The maximal isometric force was measured using hand-held dynamometry (HHD; microFET2, Procare B.V., Groningen, NED), which has been proven a valid tool for measuring strength in adults with CP [16]. Maximum force was determined from hip flexors, extensors, abductors and adductors, knee flexors and extensors, ankle dorsiflexors, and plantar flexors [7]. A *make* test, in which participants were instructed to increase muscle force gradually by pushing maximally for 5 s against the resistance given by the instructor, was employed. In total, three peak isometric forces were measured and captured for each direction and on each leg. In cases where the last trial yielded the highest score, additional trials were performed until the last trial no longer presented the highest score. Torque was calculated by multiplying the generated force with the lever arm, which was defined as the distance between the position of the HHD and the estimated joint centre of rotation. Torque values were normalized for body weight. The average of the three trials per leg was calculated. Previous research showed excellent test–retest reliability for lower limb HHD measurements in individuals with CP [18].

#### 2.2.3. Selectivity

Selectivity was determined for hip flexors, extensors, abductors and adductors, knee flexors and extensors, ankle dorsiflexors, and plantar flexors [16]. The level of selectivity was classified as; 0 (*patterned movement only*), 1 (*partially isolated movement*), and 2 (*completely isolated movement*). Previous research indicated strong agreement between test and retest in children with CP [19].

#### 2.2.4. Muscle Tone

Muscle tone was determined using the Ashworth scale [20] for hip flexors, hip adductors, knee flexors, knee extensors, and ankle plantar flexors. The classification was: 0 (*no increase in tone*), 1 (*slight increase in tone, giving a catch when the limb was moved in flexion or extension*), 2 (*more marked increase in tone, but limb could still be easily flexed or extended*), 3 (*considerable increase in tone and passive movement was difficult*), and 4 (*rigid limb in flexion or extension*). The muscle tone tests were performed at a moderate speed of about 180° per second.

#### 2.2.5. Balance

The timed up and go test (TUG) was used to assess dynamic balance [21]. Participants were asked to stand up from a standard chair, walk a distance of 3 m as fast as possible, turn around, walk back, and sit down again while wearing their normal shoes and using their normal assistive devices, if applicable. Three attempts were performed, and the best time out of these attempts was taken into account. Previous research showed good test and retest reliability of the TUG in individuals with spastic CP [22].

Standing balance was determined using the clinical test of sensory interaction on balance (CTSIB) test. Participants were asked to stand in four different conditions: (i) on a firm surface with their eyes open, (ii) on a firm surface with their eyes closed, (iii) on a foam surface with their eyes open, and (iv) on a foam surface with their eyes closed. Performance was assessed based on the time that participants maintained their balance and categorized as 0 (*unable*), 1 (*less than 30 s*), 2 (*30 s unstable*), or 3 (*30 s stable*). The CTSIB showed excellent test and retest reliability in adults with spastic CP [23].

Participants were asked to classify how often they fall (“never,” “occasionally,” “weekly,” or “daily”) when (1) standing still, (2) walking indoors, and (3) walking outdoors.

### 2.3. Statistical Analysis

The homogeneity of data was assessed with a Shapiro–Wilk test. Since most of the data were not normally distributed, data are presented as median and interquartile ranges (IQR), or percentages (%). For PROM, strength selectivity and muscle tone measures for individual legs (left and right separately) were considered. Differences in outcome measures, as noted between adults with CP (as a group) and TD adults, were investigated using a Mann–Whitney *U* test (significance: *p* < 0.05). Within adults with CP, differences in outcome measures regarding SES levels (low, middle, and high) were analyzed using a Kruskal–Wallis test and Dunn's post hoc test (Bonferroni correction: *p* = 0.05/3; *p* < 0.0167). Statistical analyses were performed using SPSS Version 20 (IBM Corp., Armonk, NY, United States), and figures were created with PRISM Version 9 (GraphPad Software, San Diego, CA, United States).

## 3. Results

Twenty-eight adults with CP and 28 matched TD adults participated in this study. The characteristics of the participants are presented in Table 1. The SES distribution for adults with CP is 10 (36%) with low SES, 9 (32%) with middle SES, and 9 (32%) with high SES. Eleven adults with CP were classified in GMFCS level I (39%), 12 in Level II (43%), and 5 in Level III (18%). No differences were noted within the CP group, per SES category, for sex (*p* = 0.630), age (*p* = 0.471), GMFCS (*p* = 0.870), and BMI (*p* = 0.874).

As presented in Table 1, adults with CP received standard care during childhood, which included a variety of orthopaedic interventions following the ISA [12].

These interventions included the Achilles tendon (*n* = 28; 100%) and hamstring lengthening (*n* = 16; 57%). Other soft tissue procedures were performed on adductors (*n* = 10; 36%), rectus femoris (*n* = 8; 29%), psoas (*n* = 6; 21%), and tibialis posterior (*n* = 3; 11%). Bony surgeries included ankle/foot corrections (*n* = 11; 39%), femoral derotation (*n* = 4; 14%), and tibial derotation (*n* = 4; 14%). In addition, the following health issues were reported: hypertension (*n* = 5, 18%), asthma (*n* = 3, 11%), diabetes (*n* = 3, 11%), incontinence (*n* = 3, 11%), and/or mental health conditions such as depression and/or anxiety (*n* = 5, 18%).

### 3.1. Range of Motion

The results of PROM assessments for adults with CP and TD adults are presented in Table 2. No differences in PROM were observed between adults with CP from different SES categories. A significantly lower PROM was observed in adults with CP when compared to TD adults in hip flexion (*p* < 0.001), hip extension (*p* = 0.029), hip abduction (*p* < 0.001), hip external rotation (*p* < 0.001), femoral anteversion (*p* = 0.007), knee flexion (*p* < 0.001), knee extension (*p* = 0.001), popliteal angels (unilateral: *p* < 0.001, bilateral: *p* < 0.001), hamstring shift (*p* = 0.002), ankle dorsiflexion (with knee extended: *p* < 0.001 and knee flexed: *p* < 0.001) and ankle plantar flexion (*p* = 0.001).

### 3.2. Strength

**Figure 1:**
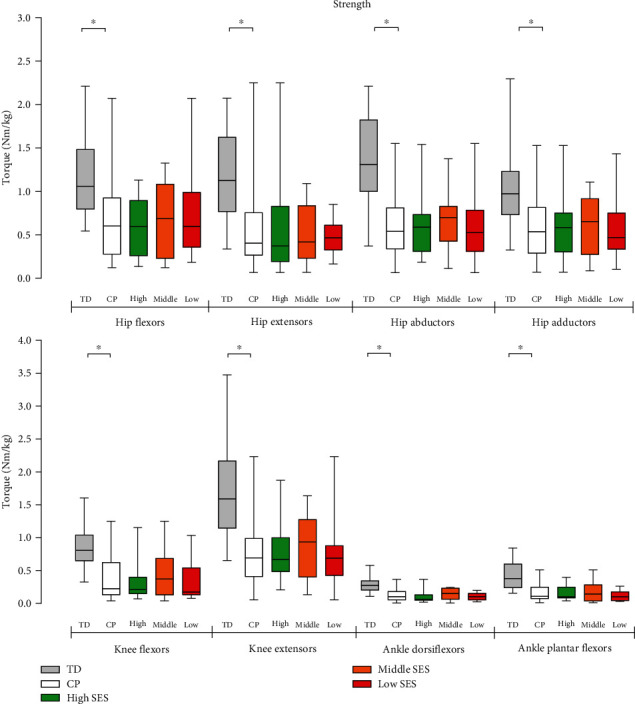
Maximal isometric torque in TD adults and adults with CP (as a group and per low, middle, and high SES category). ⁣^∗^Significantly different between adults with CP and TD adults: *p* < 0.05.

The results of maximal isometric torque tests for CP and TD adults are presented in Figure 1. With adults with CP, strength measurements for some movements were impaired by pain. Due to this, the data could *not* be obtained for extension (5 legs), knee flexion (5 legs), knee extension (1 leg), ankle dorsiflexion (10 legs), and ankle plantar flexion (7 legs). No maximal isometric torque differences were observed between adults with CP from different SES categories. The maximal isometric torque of adults with CP was significantly lower compared to TD adults (hip flexion (*p* < 0.001), hip extension (*p* < 0.001), hip abduction (*p* < 0.001), hip adduction (*p* < 0.001), knee flexion (*p* < 0.001), knee extension (*p* < 0.001), ankle dorsiflexion (*p* < 0.001), and ankle plantar flexion (*p* < 0.001)).

### 3.3. Selectivity

**Figure 2:**
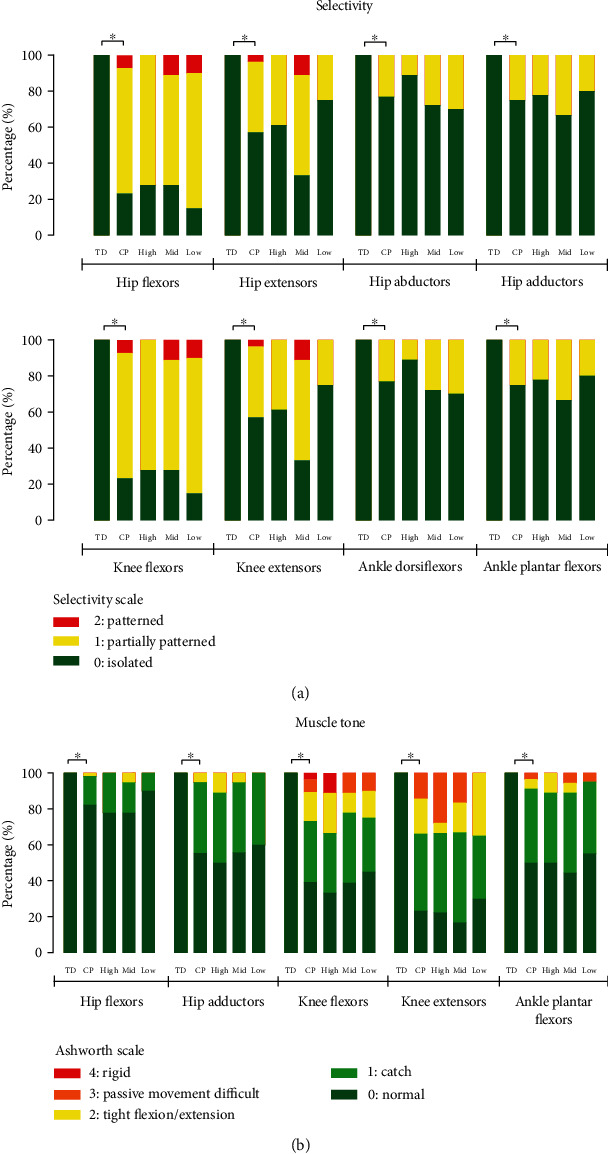
Stacked bar graphs of (a) selectivity and (b) muscle tone of TD adults and adults with CP (as a group and per low, mid (middle), and high SES category). ⁣^∗^Significantly different.

No differences in selectivity were observed in adults with CP from different SES categories. Selectivity was significantly lower in adults with CP when compared to TD adults for hip flexion (*p* < 0.001), hip extension (*p* < 0.001), hip abduction (*p* < 0.001), hip adduction (*p* < 0.001), knee flexion (*p* < 0.001), knee extension (*p* < 0.001), ankle dorsiflexion (*p* < 0.001), and ankle plantar flexion (*p* < 0.001) (Figure 2(a)).

### 3.4. Muscle Tone

There were no differences observed in muscle tone of any of the assessed muscle groups between adults with CP from different SES categories (Figure 2(b)). The muscle tone of all assessed muscle groups was significantly higher in adults with CP when compared to TD adults: hip flexors (*p* = 0.001), hip adductors (*p* < 0.001), knee flexors (*p* < 0.001), knee extensors (*p* < 0.001), and ankle plantar flexors (*p* < 0.001).

### 3.5. Balance

**Figure 3:**
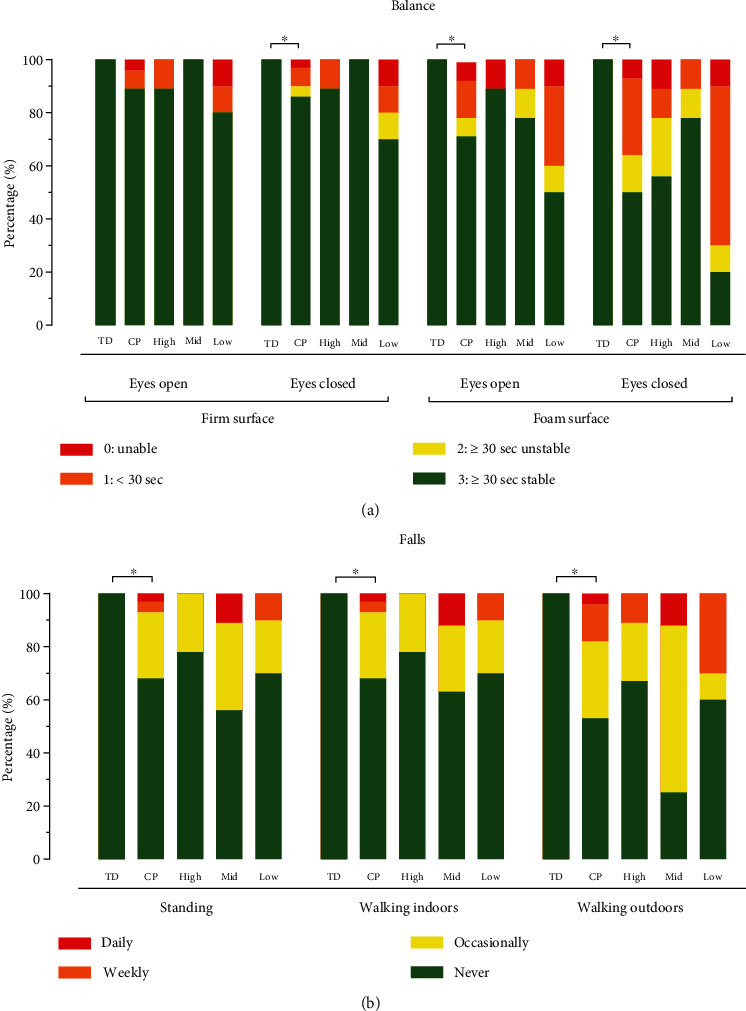
Stacked bar graphs of (a) CTSIB test and (b) falls of TD adults and adults with CP (as a group and per low, mid (middle), and high SES category). ⁣^∗^Significantly different.

Two adults with CP were not able to perform the TUG test as they could not stand up from a chair independently, and hence, these data were excluded from the analysis. No differences in TUG test and CTSIB test results were observed, however, between adults with CP from different SES categories. Adults with CP completed the TUG test significantly slower (median (IQR) = 7.6 (6.0–9.8) s) than TD adults (3.8 (3.5–4.1) s). Regarding the CTSIB test, adults with CP achieved significantly lower (standing) balance scores than TD adults on a firm surface with their eyes closed (*p* = 0.040) and on a foam surface with their eyes open (*p* = 0.003) and closed (*p* < 0.001). No differences were observed between the cohorts on the firm surface with eyes open (Figure 3(a)).

No differences were observed between the number of falls of adults with CP from different SES categories (Figure 3(b)). Adults with CP reported *falling* more frequently while standing (*p* = 0.001), walking indoors (*p* = 0.002), and outdoors (*p* < 0.001) compared to TD adults.

## 4. Discussion

The results of this study showed that no differences were observed in physical health status, including PROM, muscle strength, selective motor control, muscle tone, and balance, between ambulant adults with CP who have a low, middle, and high SES. These findings indicate that SES does not seem to influence physical health status in adults with CP who live in an urban area in SA. A secondary finding of this study was that ambulatory adults with CP presented with reduced PROM and muscle strength, lower selective motor control, increased muscle tone, loss of balance, and fall more frequently than their TD peers.

The finding that adults with CP from different SES categories present with similar physical statuses and levels of balance could be attributed to a variety of factors. Adults with CP and high SES may have better and/or more regular access to medical aid services, physiotherapy, walking aids, and transport [9]. Adults with CP with a lower SES, however, may have limited or no access to these services, which is often observed in low-to-middle-income countries where not all individuals have access to medical aid services [24]. In addition, research has shown that health inequity for individuals with disabilities still exists [25]. Most commonly, individuals with disability living in low-to-middle-income countries receive rehabilitation therapy throughout their time in school, while access to rehabilitation therapy often falls away after graduation. With limited access to medical aid services, including physiotherapy, walking aids, and transport, the reality of day-to-day living may be that individuals are required to walk longer distances and/or have to overcome more difficult obstacles during physical activity. While individuals living in high-income countries may experience fewer of these obstacles in daily life, these obstacles may positively influence physical status and balance control in adults with CP living in low-to-middle-income countries. Research studies investigating potential factors that facilitate or hamper the physical status of adults with CP living in both low-to-middle and high-income countries are, however, needed to confirm these hypotheses.

The findings that adults with CP presented with a more limited physical health status compared to TD adults are in line with findings from high-income countries [5, 26–31]. More specifically, adults with CP presented with lower PROM in most assessments across the lower limbs (13/17 assessments) in comparison to TD adults. This result is consistent with previous research by Gannotti et al. [32], who noted that adults with CP showed a reduction in PROM of hip flexion and knee extension during adulthood. In addition, previous research studies have indicated that PROM impairments are associated with ageing and closely linked to the progression of secondary conditions of CP [33]. These secondary conditions of CP could, in turn, result in reduced walking function and reduced aerobic capacity.

As expected, adults with CP showed increased muscle tone and reduced selective motor control. Previous research has shown that in children with CP, impaired selective motor control was associated with gait impairments, including increased knee flexion at initial contact and reduced step length and walking speed [34]. These secondary CP-related conditions could progress into adulthood. In addition, lower strength levels and reduced balance presented by adults with CP were consistent with previous research from high-income countries [35, 36]. These findings offer valuable insights into the health status of adults with CP in low-to-middle-income countries, enabling clinicians to implement evidence–based clinical practices. Future research is needed to investigate the development of physical health status, including muscle tone, selective motor control, strength, and balance, of individuals with CP as they grow into adulthood and beyond, specifically in low-to-middle-income countries.

The main limitation of the current study is that SES was estimated based on the housing index of the participants. However, research has shown that this can be used as a good indicator of SES [15]. Although income capital potentially would have been an even better indicator of SES, gathering this type of information is ethically debatable and is frequently not supported by many research ethics committees. In addition, the sample size in the study is rather small, while the study has specifically focussed on ambulatory adults with CP. Future research should also focus on nonambulatory adults with CP, GMFCS levels IV and V, in which the impact of SES might be different than in ambulatory adults with CP.

In conclusion, and in line with current research in developed countries, the physical health status of adults with bilateral spastic CP is poorer than that of matched TD adults. However, no differences in health status seem to exist in ambulatory adults with bilateral spastic CP with different SES backgrounds. The findings of this paper highlight that caution should be taken about the preassumption that a lower SES is always associated with a poorer health status in ambulatory adults with CP.

## Figures and Tables

**Table 1:** Demographic characteristics of the adults with CP and TD adults. Data are presented as median (IQR) or *n* (%).

**Parameter**	**CP**				**TD**
	**High SES (** **n** = 9**)**	**Middle SES (** **n** = 9**)**	**Low SES (** **n** = 10**)**	**All CP (** **n** = 28**)**	**All TD (** **n** = 28**)**
Sex (male/female)	5/4 (56/44)	3/6 (33/67)	4/6 (40/60)	12/16 (43/57)	12/16 (43/57)
Age (years)	39.7 (31.8–48.7)	42.5 (36.4–46.2)	36.7 (31.8–42.2)	39.0 (34.0–45.7)	38.5 (32.6–46.4)
GMFCS Level I/II/III	3/4/2 (33/44/22)	4/3/2 (44/33/22)	4/5/1 (40/50/10)	11/12/5 (39/43/18)	n.a.
BMI (kg/m^2^)	29.7 (23.2–31.8)	25.2 (20.5–35.8)	28.0 (23.4–31.1)	28.0 (22.8–31.5)	26.9 (23.5–29.4)
SES (housing index)	0.5 (0.5–0.8)	1.0 (1.0–1.3)	2.0 (2.0–3.1)	1.2 (0.8–2.0)	0.8 (0.7–1.1)
Orthopaedic interventions	4 (2–7)	5 (2–6)	6 (3–7)	5 (2–6)	n.a.
Surgical events	3 (2–5)	3 (2–4)	3 (2–5)	3 (2–4)	n.a.

Abbreviations: BMI, body mass index; CP, cerebral palsy; GMFCS, gross motor function classification system; IQR, interquartile ranges; n.a., not applicable; SES, socioeconomic status; TD, typically developed.

**Table 2:** Overview of passive range of motion (degrees) presented as median (IQR) for adults with CP and TD adults.

**Parameter**	**CP**				**TD**
	**High SES (** **n** = 20** legs)**	**Middle SES (** **n** = 18** legs)**	**Low SES (** **n** = 18** legs)**	**All CP (** **n** = 56** legs)**	**All TD (** **n** = 56** legs)**
Hip					
Flexion	115 (104–125)	115 (109–121)	110 (120–129)	115 (106–125)⁣^∗^	135 (121–140)⁣^∗^
Extension	20 (14–20)	15 (12–20)	20 (11–20)	20 (14–20)⁣^∗^	20 (15–25)⁣^∗^
Abduction	45 (40–51)	40 (35–46)	45 (35–49)	45 (36–50)⁣^∗^	60 (50–65)⁣^∗^
Adduction	30 (25–30)	25 (25–25)	25 (21–30)	25 (25–30)	25 (20–30)
External rotation	43 (34–51)	40 (35–46)	45 (36–50)	43 (35–50)⁣^∗^	50 (40–55)⁣^∗^
Internal rotation	55 (49–60)	55 (50–60)	60 (46–65)	55 (50–60)	53 (46–60)
Femoral anteversion	15 (12–20)	20 (12–20)	15 (11–20)	15 (12–20)⁣^∗^	15 (10–15)⁣^∗^
Knee					
Flexion	135 (125–136)	130 (121–140)	135 (130–140)	135 (125–140)⁣^∗^	145 (140–150)⁣^∗^
Extension	5 (0–10)	2 (-1–8)	0 (0–10)	5 (0–10)⁣^∗^	7 (5–10)⁣^∗^
Popliteal angle (unilateral)	35 (25–41)	40 (30–45)	35 (35–49)	35 (30–45)⁣^∗^	15 (6–20)⁣^∗^
Popliteal angle (bilateral)	20 (10–26)	28 (20–31)	20 (15–38)	20 (15–30)⁣^∗^	5 (0–10)⁣^∗^
Hamstring shift	15 (10–21)	10 (5–16)	15 (6–24)	13 (10–20)⁣^∗^	10 (5–13)⁣^∗^
Thigh to foot angle	12 (10–15)	10 (7–15)	10 (6–15)	10 (8–15)	10 (6–12)
Bimalleolar axis	15 (10–24)	14 (9–26)	20 (12–25)	15 (10–25)	15 (10–20)
Ankle					
Dorsiflexion (knee flexed)	15 (10–20)	15 (10–20)	13 (10–20)	15 (10–20)⁣^∗^	20 (20–25)⁣^∗^
Dorsiflexion (knee extended)	5 (0–10)	0 (0–5)	0 (0–9)	0 (0–7)⁣^∗^	12 (10–15)⁣^∗^
Plantar flexion	33 (30–41)	40 (30–51)	40 (31–45)	40 (30–45)⁣^∗^	45 (40–54)⁣^∗^

Abbreviations: CP, cerebral palsy; IQR, interquartile ranges; SES, socioeconomic status; TD, typically developed.

⁣^∗^Significantly different between adults with CP and TD adults: *p* < 0.05.

## Data Availability

The data that support the findings of this study are available, upon reasonable request.

## References

[1] Blair E., Langdon K., McIntyre S., Lawrence D., Watson L. (2019). Survival and mortality in cerebral palsy: observations to the sixth decade from a data linkage study of a total population register and national death index. *BMC Neurology*.

[2] Haak P., Lenski M., Hidecker M. J. C., Li M., Paneth N. (2009). Cerebral palsy and aging. *Developmental Medicine & Child Neurology*.

[3] Benner J. L., Noten S., Limsakul C. (2019). Outcomes in adults with cerebral palsy: systematic review using the international classification of functioning, disability and health. *Developmental Medicine & Child Neurology*.

[4] Jeffries L., Fiss A., Mccoy S. W., Bartlett D. J. (2016). Description of primary and secondary impairments in young children with cerebral palsy. *Pediatric Physical Therapy*.

[5] Geertsen S. S., Kirk H., Lorentzen J., Jorsal M., Johansson C. B., Nielsen J. B. (2015). Impaired gait function in adults with cerebral palsy is associated with reduced rapid force generation and increased passive stiffness. *Clinical Neurophysiology*.

[6] Eken M. M., Lamberts R. P., Koschnick S., du Toit J., Veerbeek B. E., Langerak N. G. (2020). Lower extremity strength profile in ambulatory adults with cerebral palsy and spastic diplegia: norm values and reliability for hand-held dynamometry. *Physical Medicine and Rehabilitation*.

[7] Damiano D. L., Wingert J. R., Stanley C. J., Curatalo L. (2013). Contribution of hip joint proprioception to static and dynamic balance in cerebral palsy: a case control study. *Journal of NeuroEngineering and Rehabilitation*.

[8] Korzeniewski S. J., Slaughter J., Lenski M., Haak P., Paneth N. (2018). The complex aetiology of cerebral palsy. *Nature Review Neurology*.

[9] Donald K. A., Samia P., Kakooza-Mwesige A., Bearden D. (2014). Pediatric cerebral palsy in Africa: a systematic review. *Seminars in Pediatric Neurology*.

[10] du Toit J. (2020). *Ageing with cerebral palsy after being treated with orthopaedic interval surgery approach during childhood*.

[11] Boucher N., Dumas F., Maltais D. B., Richards C. L. (2010). The influence of selected personal and environmental factors on leisure activities in adults with cerebral palsy. *Disability and Rehabilitation*.

[12] McDowell B. (2008). The gross motor function classification system--expanded and revised. *Developmental Medicine & Child Neurology*.

[13] Langerak N. G., Tam N., Du Toit J., Fieggen A. G., Lamberts R. P. (2019). Gait pattern of adults with cerebral palsy and spastic diplegia more than 15 years after being treated with an interval surgery approach: implications for low-resource settings. *Indian Journal of Orthopaedics*.

[14] World Medical Association (2013). World Medical Association Declaration of Helsinki. *Journal of the American Medical Association*.

[15] Langerak N. G., Du Toit J., Burger M., Cotton M. F., Springer P. E., Laughton B. (2014). Spastic diplegia in children with HIV encephalopathy: first description of gait and physical status. *Developmental Medicine & Child Neurology*.

[16] Novacheck T. F., Trost J. P., Sohrweide S. (2010). Examination of the child with cerebral palsy. *Orthopedic Clinics of North America*.

[17] Maltais D. B., Ferland C., Perron M., Roy J. S. (2019). Reliability of inclinometer-derived passive range of motion measures in youth with cerebral palsy. *Physical & Occupational Therapy in Pediatrics*.

[18] Crompton J., Galea M. P., Phillips B. (2007). Hand-held dynamometry for muscle strength measurement in children with cerebral palsy. *Developmental Medicine & Child Neurology*.

[19] Löwing K., Brogren Carlberg E. (2009). Reliability of the selective motor control scale in children with cerebral palsy. *Advances in Physiotherapy*.

[20] Lee K. C., Carson L., Kinnin E., Patterson V. (1989). The Ashworth scale: a reliable and reproducible method of measuring spasticity. *Neurorehabilitation and Neural Repair*.

[21] Podsiadlo D., Richardson S. (1991). The timed "up & go": a test of basic functional mobility for frail elderly persons. *Journal of the American Geriatrics Society*.

[22] Christopher A., Kraft E., Olenick H., Kiesling R., Doty A. (2021). The reliability and validity of the timed up and go as a clinical tool in individuals with and without disabilities across a lifespan: a systematic review. *Disability and Rehabilitation*.

[23] Levin I., Lewek M. D., Giuliani C., Faldowski R., Thorpe D. E. (2019). Test-retest reliability and minimal detectable change for measures of balance and gait in adults with cerebral palsy. *Gait and Posture*.

[24] Moeti T., Mokhele T., Weir-Smith G., Dlamini S., Tesfamicheal S. (2023). Factors affecting access to public healthcare facilities in the city of Tshwane, South Africa. *International Journal of Environmental Research and Public Health*.

[25] Gréaux M., Moro M. F., Kamenov K., Russell A. M., Barrett D., Cieza A. (2023). Health equity for persons with disabilities: a global scoping review on barriers and interventions in healthcare services. *International Journal for Equity in Health*.

[26] Neyroud D., Armand S., de Coulon G. (2017). Plantar flexor muscle weakness and fatigue in spastic cerebral palsy patients. *Research in Developmental Disabilities*.

[27] Opheim A., Jahnsen R., Olsson E., Stanghelle J. K. (2012). Balance in relation to walking deterioration in adults with spastic bilateral cerebral palsy. *Physical Therapy*.

[28] Nieuwenhuijsen C., van der Slot W. M. A., Dallmeijer A. J. (2011). Physical fitness, everyday physical activity, and fatigue in ambulatory adults with bilateral spastic cerebral palsy. *Scandinavian Journal of Medicine & Science in Sports*.

[29] Nieuwenhuijsen C., van der Slot W., Beelen A. (2009). Inactive lifestyle in adults with bilateral spastic cerebral palsy. *Journal of Rehabilitation Medicine*.

[30] Gjesdal B. E., Jahnsen R., Morgan P., Opheim A., Mæland S. (2020). Walking through life with cerebral palsy: reflections on daily walking by adults with cerebral palsy. *International Journal of Qualitative Studies on Health and Well-being*.

[31] Opheim A., Jahnsen R., Olsson E., Stanghelle J. K. (2009). Walking function, pain, and fatigue in adults with cerebral palsy: a 7-year follow-up study. *Developmental Medicine & Child Neurology*.

[32] Gannotti M. E., Gorton G. E., Nahorniak M. T., Masso P. D. (2013). Gait and participation outcomes in adults with cerebral palsy: a series of case studies using mixed methods. *Disability and Health Journal*.

[33] Nordmark E., Hägglund G., Lauge-Pedersen H., Wagner P., Westbom L. (2009). Development of lower limb range of motion from early childhood to adolescence in cerebral palsy: a population-based study. *BMC Medicine*.

[34] Zhou J. Y., Lowe E., Cahill-Rowley K., Mahtani G. B., Young J. L., Rose J. (2019). Influence of impaired selective motor control on gait in children with cerebral palsy. *Journal of Children's Orthopaedics*.

[35] De Groot S., Dallmeijer A., Bessems P., Lamberts M., Woude L., Janssen T. (2012). Comparison of muscle strength, sprint power and aerobic capacity in adults with and without cerebral palsy. *Journal of Rehabilitation Medicine*.

[36] Morgan P., Mcginley J. (2013). Performance of adults with cerebral palsy related to falls, balance and function: a preliminary report. *Developmental Neurorehabilitation*.

